# Development and psychometric validity of the perioperative anxiety scale-7 (PAS-7)

**DOI:** 10.1186/s12888-021-03365-1

**Published:** 2021-07-16

**Authors:** Chengjiao Zhang, Xitong Liu, Tianran Hu, Fei Zhang, Lingyi Pan, Yan Luo, Zhen Wang

**Affiliations:** 1 Shanghai Mental Health Center, Shanghai Jiao Tong University School of Medicine, Shanghai, China; 2 Department of Anesthesia and Pain Management, Ruijin Hospital, Shanghai Jiao Tong University School of Medicine, Shanghai, China

**Keywords:** Perioperative anxiety scale-7, Reliability, Validity, Localization

## Abstract

**Background:**

Preoperative anxiety is a common psychological reaction in perioperative patients. The absence of a valid measurement tool hinders the evaluation of interventions to treat preoperative anxiety in China. This study aims to develop the Perioperative Anxiety Scale-7 (PAS-7) and test its reliability, validity, and cut-off value.

**Methods:**

A total of 280 patients over 16 years old (*M* = 55.1, *SD* = 14.3) who were undergoing elective surgery were recruited to complete the PAS-7 and the Generalized Anxiety Disorder-7 scale (GAD-7) one day before surgery.

**Results:**

The PAS-7 included seven items divided into two dimensions: mental anxiety and somatic anxiety. These two dimensions could explain 74.294% of the population variance. The internal consistency of each dimension ranged from 0.761–0.933. The confirmatory factor analysis showed that the model fit of the scale was good (*χ*^*2*^= 34.798, df = 13, *χ*^*2*^/df = 2.677, CFI = 0.949, TLI = 0.918, SRMR = 0.057, RMSEA = 0.115). The correlations between the GAD-7 and each dimension and the scale’s total score were significant (0.711–0.789). A cut-off score of 8, maximizing the Youden Index, yielded a sensitivity of 75% and a specificity of 84.6% (95% *CI*: 0.88 ~ 0.97).

**Conclusions:**

The PAS-7 had good reliability and validity and could be used as an effective tool to evaluate preoperative anxiety.

**Supplementary Information:**

The online version contains supplementary material available at 10.1186/s12888-021-03365-1.

## Background

Preoperative anxiety is a common psychological reaction among perioperative patients [1]; the incidence of this reaction is high both domestically, at approximately 50% [2, 3], and abroad, at 40% ~ 80% [4, 5]. Individual trait anxiety, understanding of operation-related information, and other factors affect patients’ level of preoperative anxiety [6]. Psychological nursing and music intervention are widely used in the intervention at present [7, 8].

The high incidence of preoperative anxiety has been suggested to be associated with many adverse effects for patients, including the following: increased postoperative pain and postoperative analgesic requirements [9, 10]; increased heart rate, blood pressure, and epinephrine levels [11, 12]; increased postoperative nausea, vomiting and delirium [13, 14]; and increased recovery times and hospital stays [15, 16]. Thus, given the high frequency and adverse outcomes of preoperative anxiety, a statistically valid assessment and timely intervention for preoperative anxiety have been significant issues for anesthetists and psychologists [17].

Currently, preoperative anxiety measurement tools exist in two categories: universal anxiety scales and specific anxiety scales [18]. Universal anxiety scales include the State-Trait Anxiety Inventory (STAI) [19–21], the Self-Rating Anxiety Scale (SAS) [22, 23], and the Hamilton Anxiety Scale (HAMA) [24, 25]. These anxiety scales are widely suitable for both patients and healthy respondents; however, their limitations include low sensitivity and less assessment of preoperative anxiety. The most common specific anxiety scales are the Generalized Anxiety Disorder-7 scale (GAD-7) [26, 27] and the Amsterdam Preoperative Anxiety and Information Scale (APAIS) [28, 29]. The GAD-7 is applied simply and widely, but it has specific restrictions on the applicable population. For example, it is necessary to exclude patients with physical symptoms, and its discriminant validity is not high among elderly patients. The APAIS is specifically used to evaluate surgical patients and has been proven effective in preoperative anxiety assessment in China [30]. However, due to being developed in another country, certain cultural differences, and a lack of items related to physical anxiety, its use in China also has certain limitations.

The absence of a proper and easily applied measurement tool in preoperative settings hinders evaluating interventions to treat preoperative anxiety among patients in China. Therefore, the purpose of this study was to develop an effective scale, namely, the Perioperative Anxiety Scale-7 (PAS-7), for the assessment of mental and somatic symptoms of preoperative anxiety.

## Methods

### Participants

A total of 280 participants who underwent an elective operation under general anesthesia from March 1st, 2019 to May 31st, 2019 were recruited from a comprehensive hospital in Shanghai. The inclusion criteria were as follows: (1) older than 16 years old; (2) undergoing elective operation under general anesthesia; (3) Chinese native speakers; (4) no history of psychiatric drug use; (5) completed the scale independently or with the doctor’s help. The participants were excluded if (1) they had poor medical conditions or (2) they could not correctly understand the meaning of the scale. A total of 280 questionnaires were sent to the participants on the day before their operation, and 256 questionnaires were completed. The information on the questionnaires mainly included the medical record number, gender, age, educational background, American Society of Anesthesiologists (ASA) grade, etc. Specifically, 109 men and 147 women completed the survey, and their ages ranged from 16 to 91 years old (*M* = 55.1, *SD* = 14.3). Twenty-eight participants completed primary school or below (10.94%), 75 completed junior high school (29.30%), 78 completed special secondary or senior high school (30.47%), and 75 had a college degree or above (29.30%). According to the ASA grade, 107 were grade I (41.80%), 138 were grade II (53.91%), 20 were grade III (3.91%), and 1 was grade IV (0.39%). Informed consent was obtained from all individual participants included in the study.

### Measures

#### Generalized anxiety Disorder-7 (GAD-7)

The GAD-7 is a convenient and straightforward self-reported anxiety scale with good reliability and validity [31] widely used in scientific research and clinical practice. It has a total of seven items. Higher scores indicate more severe anxiety symptoms. Some Chinese researchers believe that the cut-off score for it in China should be adjusted to 6 points, rather than the cut-off of 10 points recommended by the developers of the scale [32, 33].

#### Perioperative anxiety Scale-7 (PAS-7)

For the PAS-7, preoperative anxiety was defined as mental and somatic anxiety among adult patients who underwent elective operation under general anesthesia. The original items of the PAS-7 were from three sources: (1) relevant references of the existing preoperative anxiety scales, such as the APAIS, GAD-7, STAI, SAS, and HAMA; (2) items from an open questionnaire survey, combined with investigation and interviews to collect the information; and (3) new items from theory structures. In the process of developing the PAS-7, a team of psychiatrists, anesthesiologists, surgeons, and other related clinical experts conducted analyses and evaluations of the structure of the scale to identify inappropriate or duplicate items and to improve the scale.

To investigate the applicability of the original items of the PAS-7, we conducted a preliminary investigation of 80 patients from a hospital in Shanghai who underwent elective operation under general anesthesia. According to the panel discussion of the initial analysis, 14 items were eventually identified in the first draft of the PAS-7. Responses are rated on a 5-point Likert scale and range from 0 (not at all) to 4 (very obvious). A higher score represents more severe preoperative anxiety. The items are shown below.

①. I’m worried about the effect of the operation.
②. I’m worried about accidents during the operation.
③. I’m worried about my life getting worse after the operation.
④. I’m worried about the pain caused by the operation.
⑤. Thinking about the operation makes me more nervous and worried than usual.
⑥. Thinking about the operation makes me easily distracted.
⑦. Thinking about the operation makes my hands tremble.
⑧. Thinking about the operation makes me lose my appetite or makes my stomach uncomfortable.
⑨. Thinking about the operation makes me use the toilet more often.
⑩. Thinking about the operation makes my face become hot and blushed, and my hands and feet sweat.
⑪. I feel fear about the operation from time to time.
⑫. I’m worried about the aftereffects of anesthesia repeatedly (such as intelligence and memory impairment).
⑬. Thinking about the surgery makes my heartbeat increasing.
⑭. Thinking about the surgery makes my breathing difficult.

### Statistical analyses

SPSS 22.0 and AMOS 22.0 were used to analyze the data as follows. (1) A correlation analysis and exploratory factor analysis were conducted by using half of the data. In the exploratory factor analysis, principal component analysis was used to extract the common factors and obtain the initial load matrix; then, VARIMAX was used to obtain the ultimate factor load matrix. The value of Kaiser-Meyer-Olkin (KMO) and Bartlett’s test were used to determine the appropriateness of the factor analysis and to perform the scree test; the number of factors was then determined based on the above results. (2) The reliability and validity of PAS-7 were conducted by using the other half of the data. The internal consistency reliability was examined by Cronbach’s *α* and the reasonable acceptability criterion of which is ≥0.70. The construct validity was examined by confirmatory factor analysis. We used the maximum likelihood method and found that the two-factor model was fitted for PAS-7 to assess the goodness-of-fit of the factor structure. Models with *χ*^*2*^ Liberty Ratio (*χ*^*2*^ / df) < 5, comparative fit index (CFI) > 0.9, Tucker-Lewis index (TLI) > 0.9, standardized root mean square residual (SRMR) < 0.1, and root mean square error of approximation (RMSEA) < 0.1 were regarded as a good fit [34]. The criterion validity was examined by the correlations between the GAD-7 and each dimension and the scale’s total score, the reasonable acceptability criterion of which is ≥0.70. (3) The Receiver-Operating Characteristic (ROC) curve was used to validate the PAS-7 against GAD-7, compare the sensitivity and specificity of PAS-7 under different cut-off scores, and determine the cut-off and predictive values of the PAS-7 in certain groups.

## Results

### Item analysis

The participants were sorted by the total score. Participants with the highest 27% of scores were defined as the high group, and those with the lowest 27% were low. The t-test revealed that there were significant differences between the two groups on all items (*p* < 0.001) (Table 1), which indicated that each item could distinguish different levels of anxiety.

**Table 1:** Item analysis of the Preoperative Anxiety Scale

Item	High group	Low group	*t*	Item	High group	Low group	*t*
1	1.710 ± 1.126	0.200 ± 0.406	7.482^***^	8	1.110 ± 0.963	0.030 ± 0.169	6.568^***^
2	1.540 ± 1.010	0.060 ± 0.236	8.475^***^	9	1.230 ± 1.165	0.030 ± 0.158	6.029^***^
3	1.400 ± 1.193	0.200 ± 0.406	5.633^***^	10	0.910 ± 1.173	0.090 ± 0.284	4.063^***^
4	2.140 ± 1.167	0.370 ± 0.490	8.281^***^	11	1.600 ± 1.063	0.060 ± 0.236	8.385^***^
5	2.200 ± 1.158	0.170 ± 0.382	9.840^***^	12	1.260 ± 0.886	0.060 ± 0.236	7.744^***^
6	1.370 ± 0.770	0.060 ± 0.236	9.654^***^	13	1.890 ± 1.105	0.030 ± 0.169	9.825^***^
7	0.710 ± 0.987	0.000 ± 0.000	4.280^***^	14	0.710 ± 0.825	0.000 ± 0.000	5.122^***^

***p<0.001

### Exploratory factor analysis

Exploratory factor analysis was carried out. Bartlett’s test showed that the KMO = 0.910, *p* < 0.001; thus, the scale was suitable for exploratory factor analysis. The scree test was also performed (see Supplementary Material for details). Through exploratory factor analysis, the authors extracted the common factors and then deleted items if any of the following criteria were met: (1) the factor loadings are close in two or more common factors; (2) only one item is under a factor; (3) the maximum factor loading is less than 0.5 on the common factor, and (4) classification is inappropriate items. Finally, using these criteria combined with the experts’ opinions, seven items were deleted, including item-3, 6, 8, 9, 11, 12, and 13. Then, exploratory factor analysis with VARIMAX was carried out on the remaining seven items. The study found two factors that explained 74.294% of the variance: F1-mental anxiety (item-1, 4, 2, 5) refers to excessive preoperatively worry and stress about the surgery and its effects, accidents, and pain; F2-somatic anxiety (item-7, 14, 10) refers to the muscle, respiratory and sensory symptoms caused by preoperative anxiety (Table 2).

**Table 2:** Exploratory factor loading matrix of the Preoperative Anxiety Scale (*n* = 128)

Item	Factors		Common degrees
	F1	F2	
1	0.848		0.739
4	0.843		0.727
2	0.818		0.763
5	0.804		0.777
7		0.859	0.774
14		0.839	0.739
10		0.786	0.682
Eigenvalue	2.879	2.321	
Contribution (%)	41.131	33.163	74.294

### Reliability analysis

Cronbach’s *α*s were calculated to measure the internal consistency of the scale. The Cronbach’s *α* was 0.933 for F1, 0.761 for F2, and 0.885 for PAS-7, showing that the scale had good internal consistency and reliability.

### Validity analysis

#### Confirmatory factor analysis

**Fig. 1:**
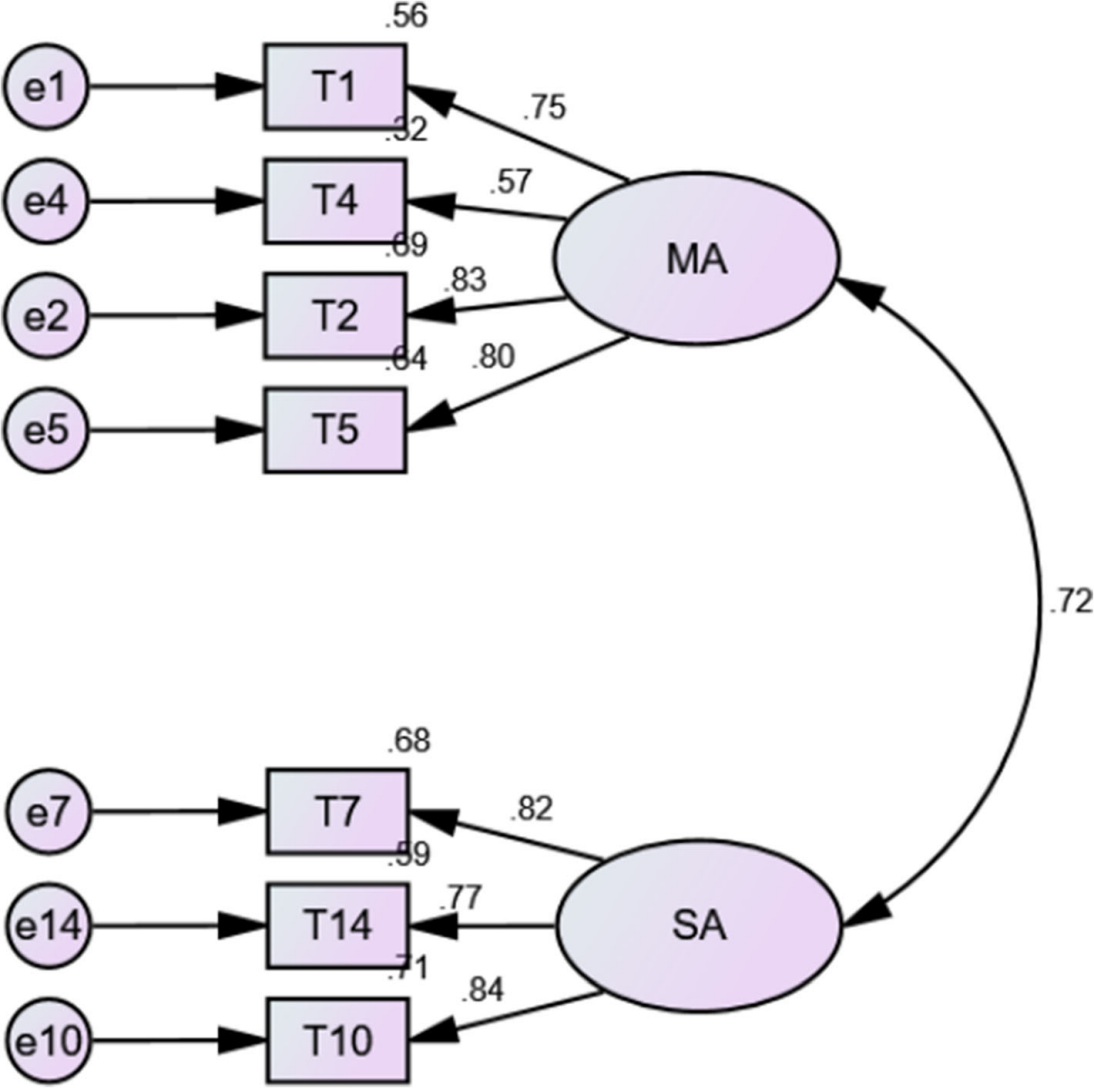
Confirmatory factor analysis path diagram for the Preoperative Anxiety Scale (*n* = 128). MA: Mental anxiety; SA: Somatic anxiety

AMOS 22.0 was used for confirmatory factor analysis, and the path diagram is shown in Fig. 1. The model fitting of the scale was ideal. In detail, the fit indices were *χ*^*2*^= 34.798, df = 13, *χ*^*2*^/df = 2.677<5, showing that the model had a good fit. CFI was 0.949 > 0.9, TLI was 0.918 > 0.9, and SRMR was 0.057 <0.1. All fit indices were acceptable. RMSEA was also acceptable at 0.115, which is nearly 0.1 (The inter-item correlation matrix was also analyzed and could be seen in Supplementary Material).

#### Criterion validity

We determined the correlation of the PAS-7 by regarding GAD-7 as the criterion. The correlation coefficients between the GAD-7 and mental anxiety, somatic anxiety, and total score of the PAS-7 were 0.711, 0.719, and 0.789 (*p* < 0.01), indicating that the PAS-7 had good criterion validity.

### The ROC curve

**Fig. 2:**
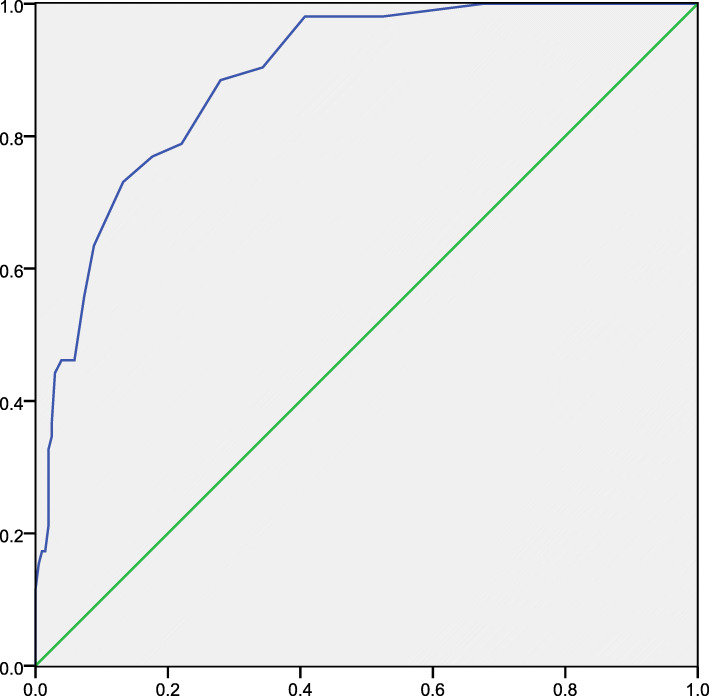
ROC curve (*n* = 256). Abscissa=1-specificity, ordinate=sensitivity

The GAD-7 score was used as a standard. We used six scores as the dividing point and divided participants into the high-anxiety group and low-anxiety group. When the Youden index was maximum, we obtained a cut-off score of 8 (with 75% sensitivity and 84.6% specificity. The area under the ROC curve (AUC) was 0.89 for the PAS-7 (95%, *CI*: 0.88 ~ 0.97) (Fig. 2).

## Discussion

This research mainly focused on developing a preoperative anxiety scale that can be widely used during preoperative evaluations in general hospitals in China (Table 3). Based on theory and research from previous scholars, the final scale includes seven items and is suitable for patients over 16 years old. Confirmatory factor analysis revealed that preoperative anxiety was divided into two dimensions: mental anxiety and somatic anxiety. Specifically, the mental anxiety factor had four items, and the somatic anxiety factor had three items. The internal consistency coefficients between the two factors ranged from 0.761–0.933, showing that the PAS-7 had good internal consistency and reliability. The ideal confirmatory factor analysis model indicated that the PAS-7 had good construct validity.

**Table 3:** Perioperative Anxiety Scale-7 (PAS-7). Instructions: This scale will assess your attitudes and feelings about your operation. Please carefully read each item, and then, according to your state in the past few days, circle the appropriate response

Item	Not at all	Some	Moderate	Relatively obvious	Very obvious
1. I’m worried about the effect of the operation.	0	1	2	3	4
2. I’m worried about accidents during the operation.	0	1	2	3	4
3. I’m worried about the pain caused by the operation.	0	1	2	3	4
4. Thinking about the operation makes me more nervous and worried than usual.	0	1	2	3	4
5. Thinking about the operation makes my hands tremble.	0	1	2	3	4
6. Thinking about the operation makes my face become hot and blushed, and my hands and feet sweat.	0	1	2	3	4
7. Thinking about the surgery makes my breathing difficult.	0	1	2	3	4

Our research adopted the GAD-7 as the criterion to evaluate the validity. We also regarded the GAD-7 as the “gold standard” for using ROC curves to determine the cut-off values, and we found that when the cut-off was 8, the PAS-7 had the largest value of screening, with a sensitivity of 75% and a specificity of 84.6%. In previous studies, the HAMA, STAI, and MINI-International Neuropsychiatric Interview [35, 36] were used as the standards to obtain cut-off values. However, there are too many items in these scales, which might impose a heavy burden on perioperative patients who suffer somatic pain. In addition, rating scales are more time-consuming for non-clinical psychological staff in general hospitals. Considering that assessments among general anesthesia patients should be short and convenient and that the general hospital lacks professionally trained evaluators, self-reported scales are the most suitable. Therefore, we adopted the GAD-7 as the criterion. Even so, the possible bias of this choice cannot be ignored. There is no consensus regarding the cut-off point for the Chinese version of the GAD-7. Although we used the cut-off value of 6 points that Chinese researchers recommended, those researchers assessed patients in the Department of Psychology of general hospitals, different from the subjects we recruited. This option might impact the currently determined cut-off value of the PAS-7. Follow-up studies could include other criteria to draw ROC curves and compare the differences to determine the best cut-off value.

Compared with other perioperative anxiety scales, the items in this study started from the core symptoms of anxiety disorders and innovatively introduced the “somatic anxiety” factor, which made anxiety assessment more complete. In patients with physical diseases, the somatic reaction to anxiety is often confused with their other physical symptoms or is easy to ignore, making treatment even more difficult. The differentiation of somatic anxiety increased the recognition of patients with preoperative anxiety, which can better prompt doctors to make corresponding treatment plans to improve such patients’ psychological feelings and prognosis [37].

Although this study established an ideal psychometric tool of perioperative anxiety, it also had some limitations. Firstly, all participants were patients who had surgery under anesthesia from the same general hospital, which might have led to selection bias, thus limiting the generalizability of our findings. Future studies should increase the sample size and increase the diversity of samples, such as patients under local anesthesia, patients undergoing ambulatory surgery, and patients using outpatient anesthesia [38]. Secondly, the sample size included in this study was limited. Not only were there not enough samples for CFA estimation, but also it was easy to cause a low fitting index, which had an impact on the results, such as RMSEA = 0.115 > 0.1. However, although it was greater than 0.1, it was very close. Considering other indexes, we concluded that the model was acceptable. Therefore, if possible, we could expand the sample size to verify again. Additionally, a previous study reported that patients with preoperative anxiety were concerned with psychological characteristics and demographic variables [39, 40]. Our study did not collect much information in this category and thus did not profoundly explore the relation between gender, education level, or other related factors. Increasing the amount of information collected from patients during clinical assessment should be considered in the future.

## Conclusion

In conclusion, this study established the Perioperative Anxiety Scale-7 and proved its validity, thus enriching this assessment tool. Compared with the current evaluation of preoperative anxiety in China, the new PAS-7 was well-targeted, easy to use, had fewer items, and needed less time. It also assessed somatic anxiety, thus making the assessment more comprehensive. In addition, the PAS-7 was suitable for Chinese patients.

## Supplementary Information

**Additional file 1.**

## Data Availability

The datasets used and analyzed during the current study are available from the corresponding author on reasonable request.

## Abbreviations

PAS-7: Perioperative anxiety scale-7
GAD-7: Generalized anxiety disorder-7 scale
APAIS: Amsterdam preoperative anxiety and information scale
STAI: State-trait anxiety inventory
HAMA: Hamilton anxiety scale
ROC: Receiver-operating characteristic curve
AUC: Area under the curve
