# Supplementary material for: Development and psychometric validity of the perioperative anxiety scale-7 (PAS-7)
Source: BMC Psychiatry. 2021 Jul 16;21:358. doi: 10.1186/s12888-021-03365-1 (PMC8284006; doi:10.1186/s12888-021-03365-1)
Supplement: Supplementary file 1 — Additional file 1. [file 12888_2021_3365_MOESM1_ESM.docx]

**scree plot**

**
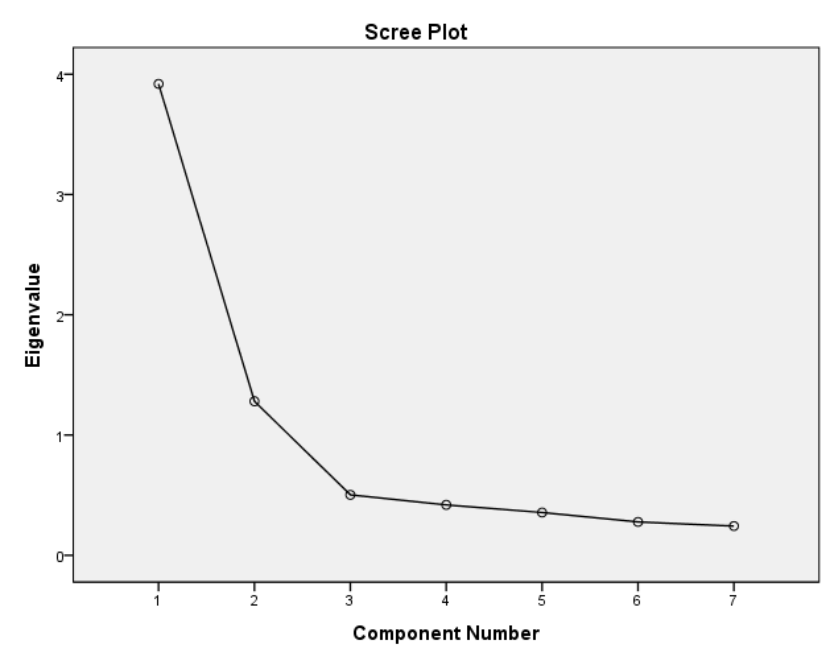
**

**an inter-item correlation matrix with mean and standard deviation values of each item**

| Descriptive statistics | | | |
| --- | --- | --- | --- |
|  | Mean | SD | N |
| T1 | 0.66 | 0.880 | 128 |
| T2 | 0.48 | 0.868 | 128 |
| T4 | 0.94 | 0.920 | 128 |
| T5 | 0.78 | 1.003 | 128 |
| T7 | 0.25 | 0.687 | 128 |
| T10 | 0.27 | 0.818 | 128 |
| T14 | 0.23 | 0.642 | 128 |

| **inter-item correlation matrix** | | | | | | | | |
| --- | --- | --- | --- | --- | --- | --- | --- | --- |
|  | | T1 | T2 | T4 | T5 | T7 | T10 | T14 |
| T1 | Pearson | 1 | 0.709^**^ | 0.392^**^ | 0.557^**^ | 0.384^**^ | 0.361^**^ | 0.357^**^ |
|  | *p* |  | 0.000 | 0.000 | 0.000 | 0.000 | 0.000 | 0.000 |
|  | N | 128 | 128 | 128 | 128 | 128 | 128 | 128 |
| T2 | Pearson | 0.709^**^ | 1 | 0.472^**^ | 0.628^**^ | 0.417^**^ | 0.444^**^ | 0.444^**^ |
|  | *p* | 0.000 |  | 0.000 | 0.000 | 0.000 | 0.000 | 0.000 |
|  | N | 128 | 128 | 128 | 128 | 128 | 128 | 128 |
| T4 | Pearson | 0.392^**^ | 0.472^**^ | 1 | 0.471^**^ | 0.349^**^ | 0.292^**^ | 0.408^**^ |
|  | *p* | 0.000 | 0.000 |  | 0.000 | 0.000 | 0.001 | 0.000 |
|  | N | 128 | 128 | 128 | 128 | 128 | 128 | 128 |
| T5 | Pearson | 0.557^**^ | 0.628^**^ | 0.471^**^ | 1 | 0.628^**^ | 0.588^**^ | 0.527^**^ |
|  | *p* | 0.000 | 0.000 | 0.000 |  | 0.000 | 0.000 | 0.000 |
|  | N | 128 | 128 | 128 | 128 | 128 | 128 | 128 |
| T7 | Pearson | 0.384^**^ | 0.417^**^ | 0.349^**^ | 0.628^**^ | 1 | 0.696^**^ | 0.619^**^ |
|  | *p* | 0.000 | 0.000 | 0.000 | 0.000 |  | 0.000 | 0.000 |
|  | N | 128 | 128 | 128 | 128 | 128 | 128 | 128 |
| T10 | Pearson | 0.361^**^ | 0.444^**^ | 0.292^**^ | 0.588^**^ | 0.696^**^ | 1 | 0.651^**^ |
|  | *p* | 0.000 | 0.000 | 0.001 | 0.000 | 0.000 |  | 0.000 |
|  | N | 128 | 128 | 128 | 128 | 128 | 128 | 128 |
| T14 | Pearson | 0.357^**^ | 0.444^**^ | 0.408^**^ | 0.527^**^ | 0.619^**^ | 0.651^**^ | 1 |
|  | *p* | 0.000 | 0.000 | 0.000 | 0.000 | 0.000 | 0.000 |  |
|  | N | 128 | 128 | 128 | 128 | 128 | 128 | 128 |
| Note: ** Correlation is significant at the 0.01 level (2-tailed). | | | | | | | | |
